# Global research trends in renal anemia: a multidimensional bibliometric study

**DOI:** 10.1080/0886022X.2025.2580457

**Published:** 2025-11-04

**Authors:** Yuanchen Niu, Yufang Wang, Changhong Huo, Yi Fang

**Affiliations:** ^a^ Key Laboratory of Molecular Target & Clinical Pharmacology and the State Key Laboratory of Respiratory Disease, School of Pharmaceutical Sciences & the Fifth Affiliated Hospital, Guangzhou Medical University, Guangzhou, China; ^b^ Phase I Clinical Research Center, Key Laboratory of Biological Targeting Diagnosis, Therapy and Rehabilitation of Guangdong Higher Education Institutes, The Fifth Affiliated Hospital of Guangzhou Medical University, Guangzhou, China; ^c^ School of Pharmaceutical Sciences, Hebei Medical University, Shijiazhuang, China; ^d^ Clinical Trial Institution Research Ward, Peking University People’s Hospital, Beijing, China

**Keywords:** Renal anemia, chronic kidney disease, bibliometric analysis, CiteSpace, VOSviewer

## Abstract

Renal anemia, a major complication of chronic kidney disease, contributes to increased cardiovascular risk, mortality, and accelerated progression to kidney failure. However, comprehensive bibliometric analyses evaluating global trends, knowledge gaps, and emerging research hotspots in this field remain lacking. This study retrieved publications related to renal anemia from the Web of Science Core Collection (January 1965–November 2024) and conducted data analysis and visualization using VOSviewer, CiteSpace, Pajek, and Origin. The final dataset included 1,664 publications authored by 7,534 researchers from 2,287 institutions across 72 countries, with Japan, the United States, and China accounting for 45.8% of total publications. Top institutions were Showa University, the University of Tokyo, and King’s College Hospital; Iain C. Macdougall was the most prolific author, while Masaomi Nangaku demonstrated the highest citation impact and centrality. Keyword analysis revealed a growing focus on clinical trials and pathophysiology, highlighting three core therapeutic strategies: erythropoiesis-stimulating agents, iron supplementation, and hypoxia-inducible factor prolyl hydroxylase inhibitors (HIF-PHIs), along with several emerging research topics targeting sodium-glucose cotransporter 2 (SGLT2), hepcidin, fibroblast growth factor 23 (FGF23), gut microbiota, zinc, and stem cells. Our conclusions indicate that the field has evolved from erythropoietin replacement therapy to HIF-PHI-centered physiological modulation. Current hotspots center on HIF-PHI clinical translation, with potential therapeutic avenues including SGLT2 inhibitors, hepcidin antagonists, FGF23 signaling modulation, gut microbiota regulation, zinc supplementation, and stem cell-based therapeutics.

## 1. Introduction

Chronic kidney disease (CKD) affects approximately 700 million adults globally, representing a significant public health challenge. Due to population aging and increasing rates of diabetes and hypertension, the global CKD burden is projected to rise further [1]. Renal anemia, a common complication of CKD, is strongly correlated with disease progression. In the United States (US), anemia affects 15.4% of CKD patients overall, with prevalence increasing from 8.4% in stage 1 to 53.4% in stage 5 [2]. Asian populations bear an even greater burden, with a 42% anemia prevalence among CKD patients [3]. This complication significantly impairs quality of life and is strongly associated with increased risks of hospitalization, cardiovascular events, renal function decline, cognitive impairment, and all-cause mortality. The development of renal anemia involves multiple underlying mechanisms, such as insufficient erythropoietin (EPO) production, impaired iron metabolism, chronic inflammation, shortened erythrocyte survival, and other factors [4]. This mechanistic complexity poses major challenges for developing standardized therapies.

For decades, researchers have sought to maintain hemoglobin levels within a therapeutic range that alleviates symptoms while preserving quality of life. Beyond erythrocyte transfusions and iron supplementation, therapeutic strategies such as EPO replacement or stimulation of endogenous EPO production have been developed. Despite their efficacy, treatment outcomes remain suboptimal in some cases. In recent years, renal anemia has garnered growing research interest, with a substantial increase in related publications. Although reviews on this topic have been published from various perspectives [4–11], traditional narrative reviews may be insufficient to capture the rapidly evolving landscape, which highlights the need for advanced analytical approaches to map research trajectories, identify knowledge clusters, and reveal emerging trends.

Bibliometric analysis serves as an effective tool for mapping and interpreting scholarly landscapes by applying mathematical and statistical methods to bibliographic data. This methodology allows researchers to transform fragmented academic outputs into structured insights, facilitating the identification of intellectual trajectories, collaboration networks, and conceptual evolution within disciplines [12,13]. However, to our knowledge, no comprehensive bibliometric studies have been conducted in the field of renal anemia. In this study, we performed a multidimensional bibliometric analysis of renal anemia research, aiming to systematically outline global research trends, identify research hotspots, and highlight future directions in this field.

## 2. Data and methods

### 2.1. Data sources and search strategies

The Web of Science Core Collection (WOSCC) was employed as the data source for this bibliometric analysis due to its rigorous journal selection, extensive historical records (since 1900), and strong compatibility with analytical tools such as CiteSpace and VOSviewer, making it a well-established and reliable database for such research [13,14]. A systematic and precise search strategy was developed to ensure comprehensive retrieval of renal anemia literature. The process commenced with consulting controlled vocabulary terms, including the Medical Subject Headings descriptor ‘Anemia, Renal’. Key synonyms and variant expressions (e.g., ‘uremic anemia’, ‘anemia of chronic kidney disease’, ‘renal anemia’, ‘renal anemic’, ‘kidney anemia’, and ‘nephrogenic anemia’) were then incorporated based on a thorough review of major clinical guidelines (e.g., KDIGO) and influential publications. To account for American/British English spelling variations, wildcard operators were employed, for example using ‘an*emia’ to retrieve both ‘anemia’ and ‘anaemia’. Exact phrase searching (enabled by quotation marks) was applied to increase specificity. All synonymous terms were combined using the Boolean OR operator to maximize recall while maintaining high precision. The strategy was further refined through iterative pre-searches and examination of the titles, abstracts, and keywords of known relevant articles. On 6 November 2024, the final search was executed in WOSCC using the query: TS = (‘renal an*emia’ OR ‘renal an*emic’ OR ‘uremic an*emia’ OR ‘kidney an*emia’ OR ‘nephrogenic an*emia’ OR ‘an*emia of chronic kidney disease’). Table S1 lists all included keywords, synonyms, truncations, and Boolean operators. Inclusion criteria were English-language articles or reviews directly relevant to renal anemia; exclusion criteria were: (1) non-English publications; (2) non-article/review documents (e.g., meeting abstracts, editorials, letters, book chapters, proceeding papers, and early access materials); and (3) corrections, reprints, and retracted publications. The search and selection process is outlined in Figure S1. All retrieved records were exported in plain text format with complete bibliographic details and cited references. To assess journal quality, we extracted the H-Index, Journal Citation Reports (JCR) data, and 2024 impact factors (IFs) for the included publications.

### 2.2. Data cleaning and standardization

The process combined automated tools and manual verification to ensure data quality: (1) Keyword merging: Synonyms, plural forms, and spelling variants (e.g., ‘renal anemia’/’renal anaemia’, ‘kidney failure’/’renal failure’) were standardized using a semi-automatic approach. We created a custom thesaurus file to map equivalent terms and applied it via VOSviewer’s term processing functionality. All high-frequency terms underwent manual review to ensure consistency. (2) Author name disambiguation: Potential name variants were identified using co-authorship and affiliation-based algorithms in CiteSpace. Results were manually verified, especially for prolific authors, to ensure accurate attribution. (3) Institutional/country standardization: Affiliations from England, Scotland, Wales, and Northern Ireland were unified under ‘UK’; those in Taiwan, Hong Kong, and Macao were consistently recorded as ‘China’; and ‘Turkey’ was updated to ‘Türkiye’. This process applied string matching with manual validation for ambiguous cases.

### 2.3. Data analysis and visualization

We employed multiple analytical tools: VOSviewer for collaboration networks; VOSviewer and Pajek for cluster analysis and visualizing the temporal evolution of keyword co-occurrence networks; CiteSpace for co-cited reference analysis, citation burst detection for references and keywords, and dual-map overlays; Origin for plotting publication trends. Table S2 outlines the tasks performed by each software tool. Figure S2 presents the detailed methodological workflow of the bibliometric analysis. Raw datasets are available in the Supplementary Files.

## 3. Results

### 3.1. Publication trends

**Figure 1.**
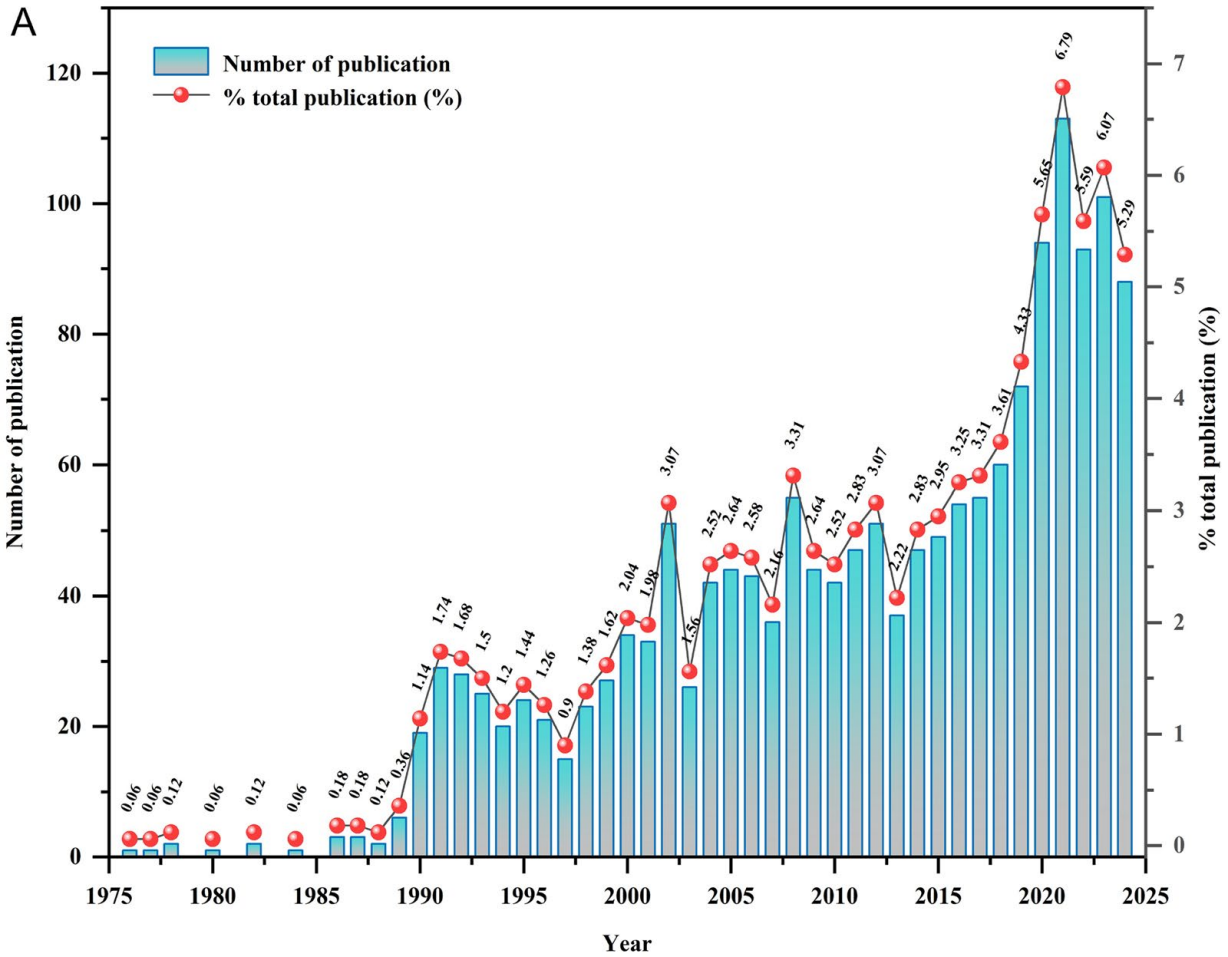
Annual publication trends in renal anemia research (1976–2024).

Our search identified 1,664 relevant publications (1,375 articles; 289 reviews) published between 1 January 1965 and 6 November 2024, with the earliest record dating to 1976 (Figure 1). Temporal analysis revealed three distinct research phases. The nascent phase (1976–1989) showed minimal output, with only 22 publications total. The fluctuating growth phase (1990–2017) exhibited gradual expansion accompanied by cyclical variations, with an average of 36.5 publications per year. The rapid growth phase (2018–2024) accounted for 37.3% of the total output, with an average of 89 publications per year and a peak of 113 in 2021. Notably, the 2024 data (88 publications) reflect partial output due to the November cutoff.

### 3.2. Country and institution analysis

**Figure 2.**
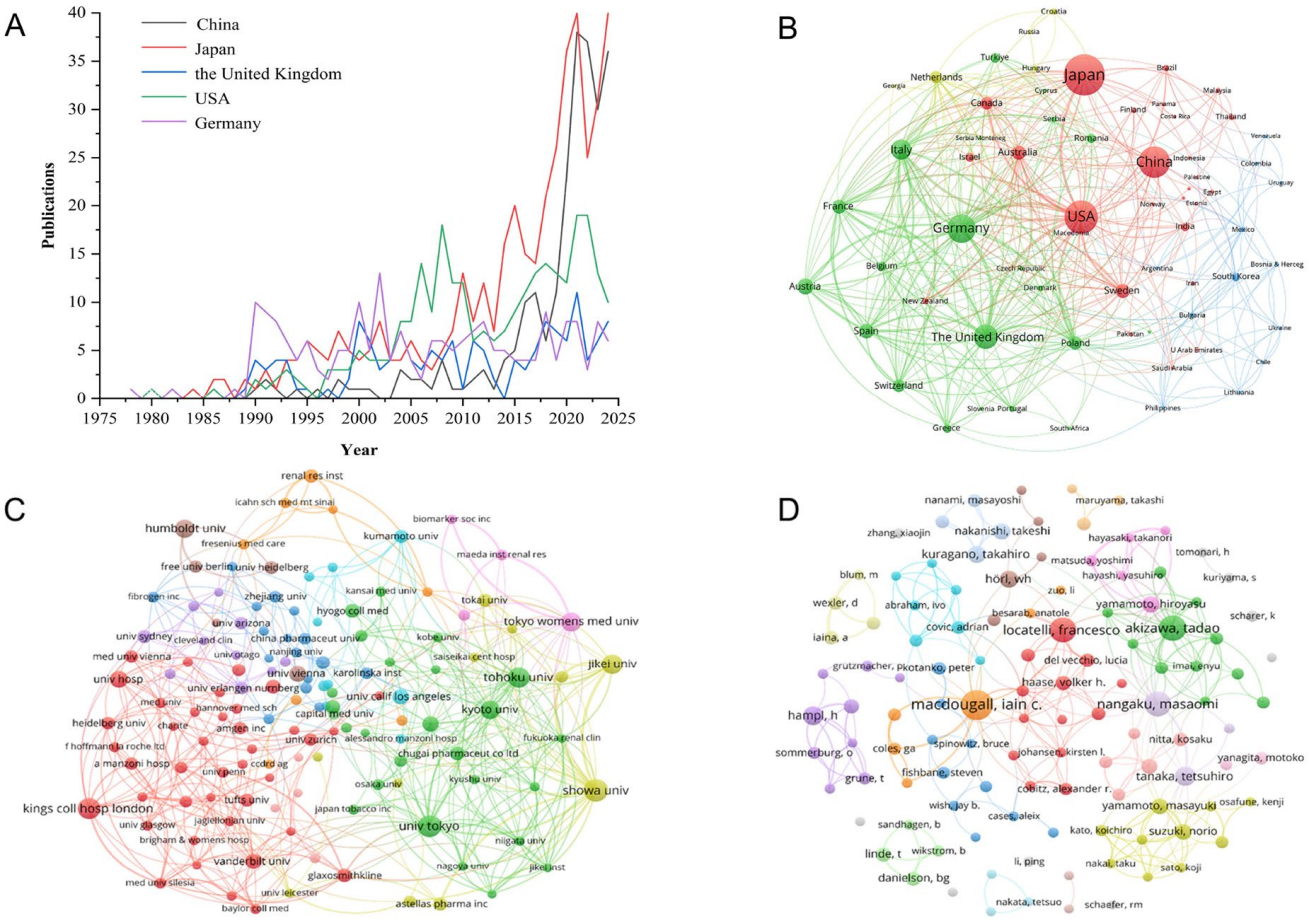
(A) Top five countries’ publication trends. (B) Country collaboration network. (C) Institutional collaboration network. (D) Author collaboration network. Node size represents the publication volume for each entity; line thickness represents the collaboration strength between them..

Our analysis identified publications from 72 countries and 2,287 institutions. Table S3 presents the top 10 most productive countries, predominantly from Europe (*n* = 6) and Asia (*n* = 2). Japan led with 407 publications (24.46% of total output), followed by the US (278; 16.71%), China (244; 14.66%), Germany (197; 11.84%), the United Kingdom (UK) (149; 8.95%), and Italy (103; 6.19%). Annual publication trends (Figure 2(A)) reveal accelerated growth in Japan and China over the past decade. Citation analysis showed the US with the highest total citations (10,433), followed by Japan (8,158), Germany (7,001), the UK (4,513), Italy (2,653), and China (2,516). Crucially, centrality metrics revealed pivotal ‘bridge’ roles in international collaboration networks [13]. The US (centrality = 0.26) and Germany (centrality = 0.17) function as dominant hubs, exhibiting the highest centrality values, indicating their capacity to connect disparate research communities. This structural advantage correlates strongly with research impact: the US and Germany had the highest average citations per publication (37.53 and 35.54, respectively), significantly exceeding Japan’s (20.04) despite its leading productivity.

The country collaboration network (Figure 2(B)) displays a polycentric structure, with these six nations forming tightly interconnected hubs. Total link strength (TLS) (Table S3) [15,16] quantifies connection robustness (higher values indicate stronger ties), further validating the centrality findings. The US holds the highest TLS (260), confirming its role as the primary conduit for international cooperation. Notably, strong TLS correlates with citation impact: nations with TLS >200 (US, Germany, and UK) averaged 35.17 citations per publication, while those with TLS <100 (e.g., Japan = 66, China = 40) averaged only 18.78 citations per publication.

Institutional analysis revealed geographic clustering: Japanese institutions accounted for six of the top 10 positions (Table S3), consistent with Japan’s national productivity. Showa University led with 33 publications, followed by the University of Tokyo (31) and King’s College Hospital (28). The University of Tokyo’s distinctly high centrality (0.07) and substantially elevated citation impact (64.61 citations per publication) positioned it as the primary institutional bridge within the network, exhibiting triple the citation rate of Showa University (20.73) despite comparable output. The remaining institutions exhibited varied regional collaboration patterns. The collaboration network among 144 institutions (≥5 publications each; Figure 2(C)) demonstrated active international partnerships alongside persistent regional clustering. Notably, eight of the top 10 institutions collaborated with pharmaceutical companies (e.g., F. Hoffmann-La Roche Ltd., Astellas Pharma Inc., and Amgen Inc.), with Humboldt University and the University of Vienna as exceptions.

### 3.3. Author analysis

A total of 7,534 authors contributed to renal anemia research. As shown in Table S4, authors from Japanese institutions held six of the top 10 most productive positions. Iain C. Macdougall ranked first with 38 publications and 1,701 total citations, while Tadao Akizawa followed with 29 publications. Notably, Masaomi Nangaku achieved the highest average citation rate (65.21) and total citations (1,826) among the leading authors, despite ranking third in publication count (28). Francesco Locatelli also ranked third in publication count (28) and had the highest H-index (90). Centrality analysis revealed limited roles as collaborative bridges across the cohort, with scores ranging from 0 to 0.03. Masaomi Nangaku demonstrated the highest centrality (0.03) among the top authors, followed by Iain C. Macdougall, Tadao Akizawa, and Hiroyasu Yamamoto (all 0.02). The author collaboration network (Figure 2(D)) revealed partnerships among 115 authors with ≥5 publications each. These leading scholars not only had high publication volumes but also maintain active collaborative relationships. Their influence is further evidenced by contributions to clinical practice guidelines: Francesco Locatelli, Iain C. Macdougall, and Walter H. Hörl helped develop European guidelines for anemia management [17], while Tadao Akizawa and Hiroyasu Yamamoto contributed to the Japanese Society for Dialysis Therapy guidelines for renal anemia [18].

### 3.4. Journal analysis

The retrieved studies on renal anemia were published in 5,912 academic journals. As presented in Table S5, *Nephrology Dialysis Transplantation* leads in publication volume (116 articles), followed by *Clinical Nephrology* (61 articles) and *Kidney International* (42 articles). Among these leading journals, only three have an IF ≥5: namely, *Kidney International* (IF 14.8), *Journal of the American Society of Nephrology* (IF 10.2), and *American Journal of Kidney Diseases* (IF 9.4). The journal quality distribution reveals that 45% rank in JCR Q1 and 35% in Q2, reflecting their specialized authority in this field. Researchers seeking current developments in renal anemia should focus on these high-impact publications.

The co-citation phenomenon occurs when two journals are cited together in subsequent studies, indicating a potential intellectual relationship between them [19]. Among the top 20 co-cited journals, eight have an IF ≥10, and 75% of these eight journals are ranked in JCR Q1 (Table S6). *Nephrology Dialysis Transplantation* (IF 4.8, JCR Q1) received the highest number of citations (4,230), closely followed by *Kidney International* (3,984), reflecting their significant influence in the field.

**Figure 3.**
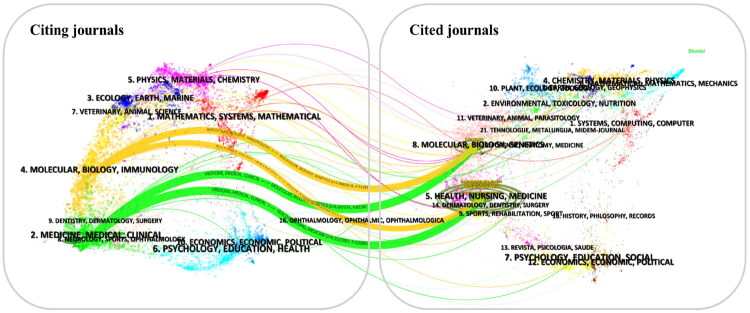
The dual-map overlay of journals. The colored trajectories represent citation flows (i.e., arcs connecting citing and cited journal clusters), with their thickness indicating the strength or frequency of citation relationships.

The dual-map overlay (Figure 3), generated using CiteSpace [20], visualizes cross-disciplinary citation patterns in renal anemia research. The left-side base map represents citing journals, which publish articles actively referencing others, reflecting current research frontiers. The right-side base map represents cited journals, which publish foundational articles cited by newer studies [21]. Prominent green and orange trajectories reveal that research from ‘health, nursing, and medicine’ and ‘molecular, biology, and genetics’ fields is frequently cited by journals in ‘medicine, medical/clinical’ and ‘molecular biology/immunology’. This pattern demonstrates the current research dominance of clinical medicine and molecular biology/immunology in renal anemia studies and the field’s interdisciplinary nature. The overlay highlights topic evolution from basic mechanisms to clinical applications.

### 3.5. Reference analysis

Table S7 presents the 20 most frequently co-cited references in renal anemia research, each with over 60 co-citations. These include a review of anemia mechanisms in CKD [22], a review of hypoxia-inducible factor prolyl hydroxylase inhibitors (HIF-PHIs) [23], a clinical practice guideline for renal anemia [24], and an observational study linking anemia to cardiovascular outcomes in end-stage renal disease (ESRD) [25]. The remaining 16 references represent pivotal clinical trials: two pioneering recombinant human erythropoietin (rHuEpo) trials [26,27]; four epoetin alfa efficacy studies [28–31]; one epoetin beta safety evaluation [32]; two darbepoetin alfa investigations [33,34]; six roxadustat randomized trials [35–40]; and one daprodustat pharmacokinetic study [41]. The most co-cited reference (241 co-citations) was the seminal *New England Journal of Medicine* publication by Singh et al. [30], which demonstrated that targeting higher hemoglobin levels (13.5 g/dL) with epoetin alfa, compared to a conservative target (11.3 g/dL), significantly increased risks of all-cause mortality, myocardial infarction, congestive heart failure, and stroke. These findings defined an evidence-based therapeutic window of 11.0–12.0 g/dL for renal anemia management, which fundamentally informed subsequent clinical guidelines [24].

**Figure 4.**
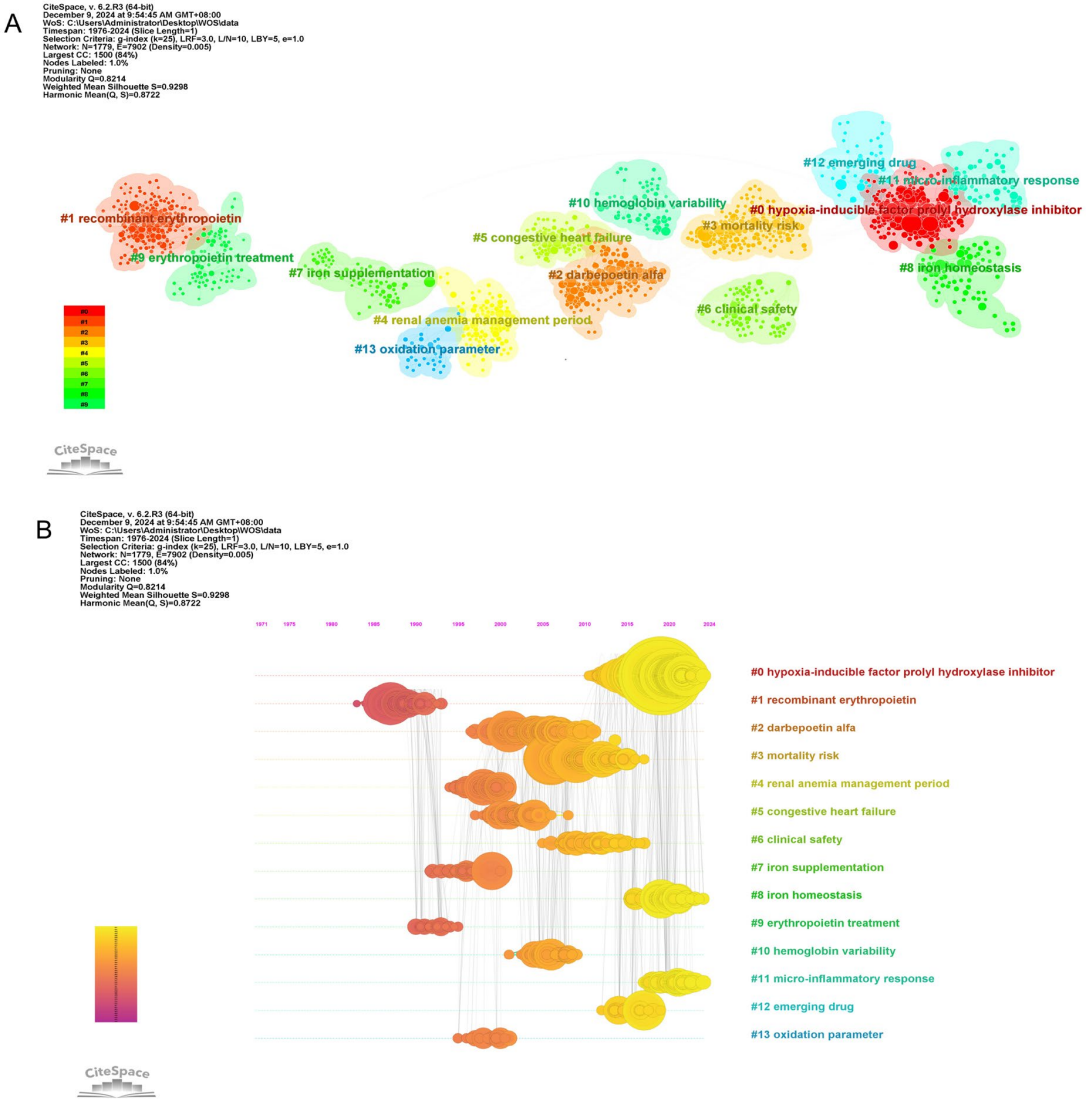
Cluster analysis (A) and temporal evolution (B) of co-cited references. Node size and color represent the total citation count and the time slice, respectively.

Co-citation cluster analysis provides valuable insights into evolving research trends. As shown in Figure 4(A), CiteSpace’s log-likelihood ratio algorithm identified 14 major keyword-based clusters, demonstrating high reliability (modularity score, *Q* = 0.8722; silhouette score, *S* = 0.9298). Notably, clusters with smaller numerical labels indicate larger research scales. The timeline visualization in Figure 4(B) illustrates the chronological development of these 14 clusters: cluster #0 (HIF-PHI) emerged as both the largest and most recent, underscoring its current research importance. This cluster shows particularly strong co-citation linkages with two other emerging areas: #8 (iron homeostasis) and #11 (micro-inflammatory response).

**Figure 5.**
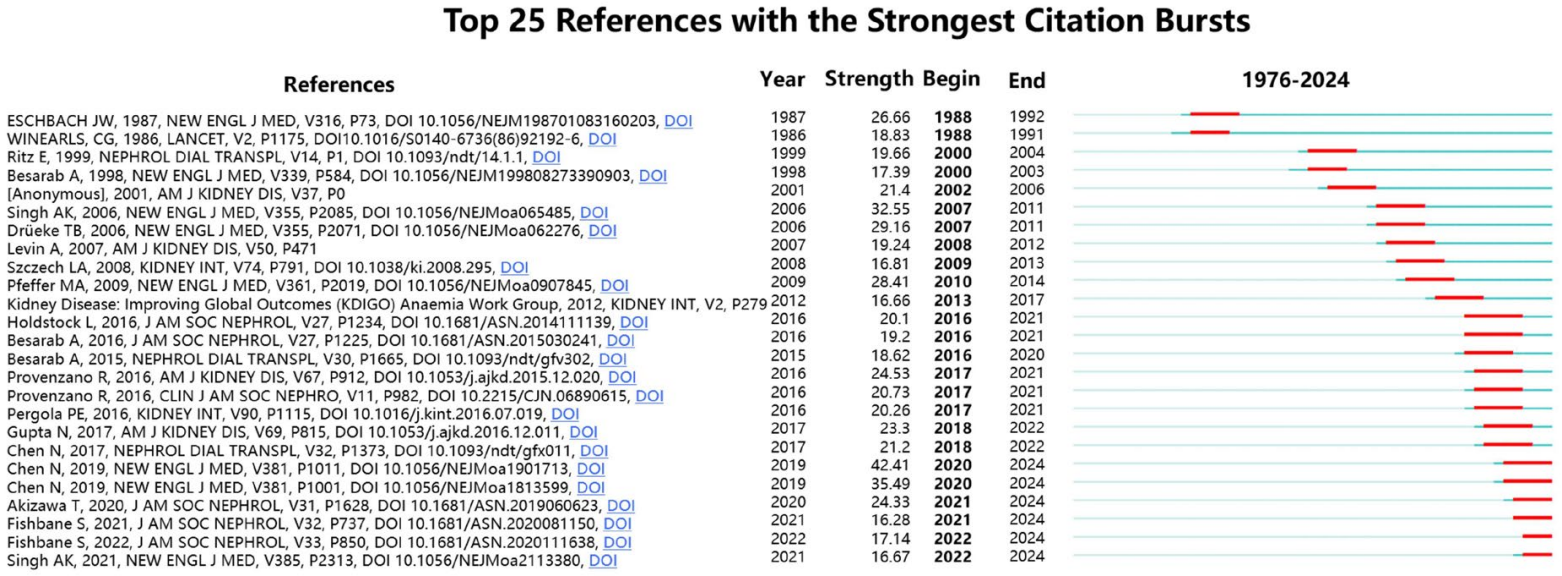
Top 25 references exhibiting the strongest citation bursts. The thick blue line represents the publication’s baseline citation period, while the thick red line represents its citation burst period.

Figure 5 displays the top 25 references with the most significant citation bursts, where red bars indicate active citation periods. Analysis reveals that the most prominent bursts correspond to two Chinese clinical trials investigating roxadustat’s therapeutic efficacy for renal anemia [38,39]. Temporal analysis further shows six references sustained citation peaks through 2024, including five roxadustat trials [38–40,42,43] and one daprodustat trial [44].

### 3.6. Keyword analysis

**Figure 6.**
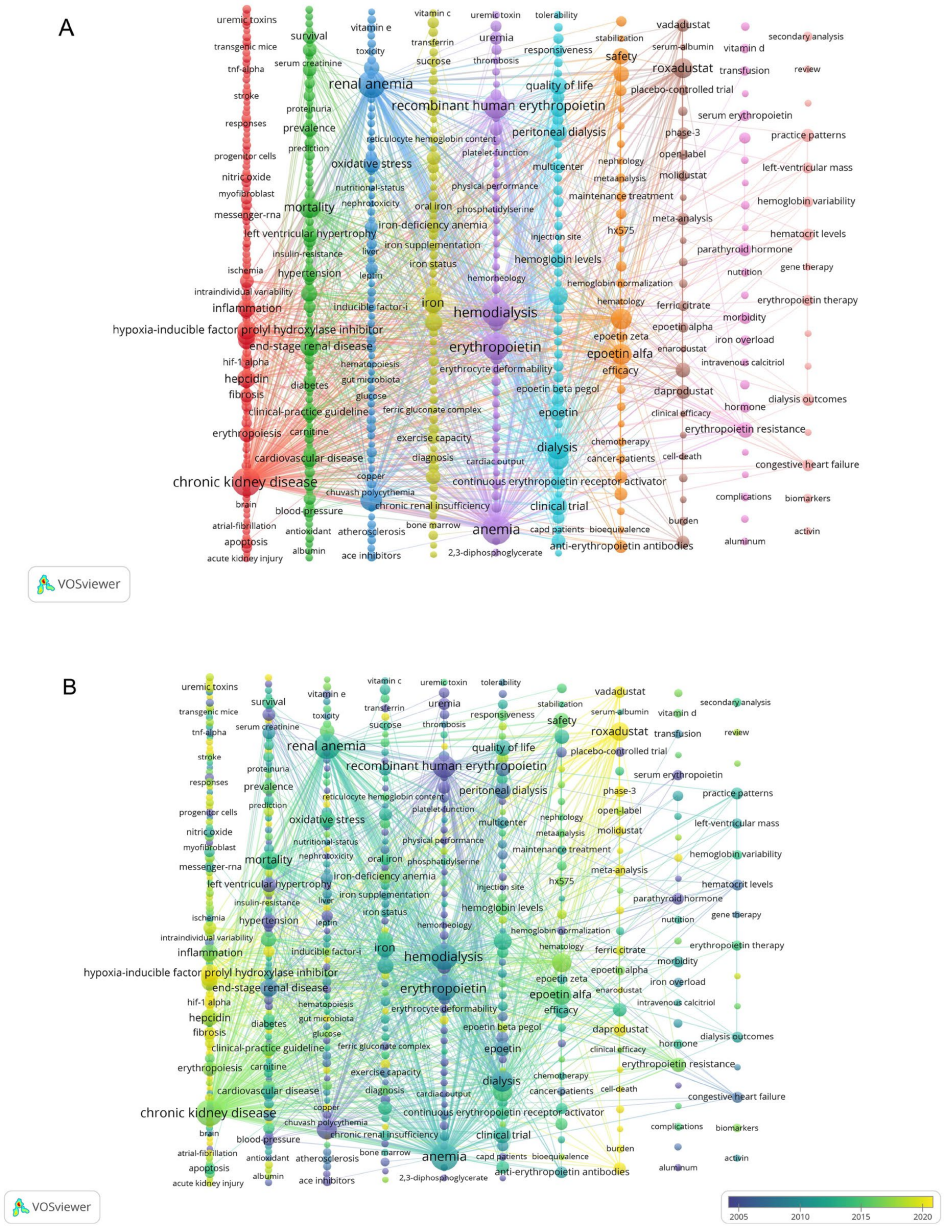
(A) Keyword co-occurrence network and cluster analysis. (B) Temporal evolution of keyword co-occurrence. Node size represents the frequency of a keyword’s appearance in the entire literature corpus; the links between nodes represent co-occurrence relationships between keywords.

Keyword co-occurrence analysis provides insights into research hotspots and trends. From 4,097 screened keywords, the top 30 high-frequency terms in renal anemia research were identified (Table S8). ‘Anemia’ ranked highest in both frequency (736) and TLS (5,275), followed by ‘hemodialysis’ (667; TLS = 4,902) and ‘CKD’ (615; TLS = 4,801). Further, 503 keywords (frequency ≥3) underwent VOSviewer-based clustering, forming 10 thematic groups. As shown in the visual clustering map (Figure 6(A)), keyword nodes were automatically categorized by semantic relevance. Cluster #1 (red): Focused on HIF pathway mechanisms, with keywords including ‘HIF-PHI’, ‘erythropoiesis’, ‘inflammation’, ‘hepcidin’, and ‘uremic toxins’. Cluster #2 (green): Explored the link between renal anemia and cardiovascular complications, incorporating terms like ‘sodium-glucose cotransporter 2 (SGLT2) inhibitors’ and ‘fibroblast growth factor 23 (FGF23)’. Cluster #3 (blue): Addressed oxidative stress and metabolic dysregulation in renal anemia pathogenesis, with terms such as ‘gut microbiota’ and ‘zinc supplementation’. Cluster #4 (yellow): Centered on optimizing iron therapy, featuring ‘oral iron’ and ‘intravenous iron therapy’. Cluster #5 (purple): Investigated red blood cell physiology and pathology. Cluster #6 (light blue): Examined erythropoiesis-stimulating agent (ESA) development and clinical application, including ‘darbepoetin alfa’ and ‘continuous erythropoietin receptor activator (CERA)’. Cluster #7 (orange): Assessed biosimilars (e.g., ‘HX575’, ‘epoetin zeta’) in renal anemia treatment. Cluster #8 (brown): Highlighted HIF-PHI clinical applications, with recent approvals like ‘roxadustat’, ‘daprodustat’, ‘vadadustat’, ‘enarodustat’, and ‘molidustat’. Cluster #9 (pink): Focused on secondary metabolic disorders in renal anemia; related keywords included ‘hyperparathyroidism’, ‘hyperphosphatemia’, and ‘phosphate binder’. Cluster #10 (light pink): Emphasized renal anemia treatment strategies. Time-overlap analysis of keywords (Figure 6(B)) revealed that early research (dark nodes) prioritized rHuEpo, while contemporary studies (bright nodes) shifted to HIF pathway modulation and iron utilization optimization. Notably, recent popular keywords like ‘SGLT2’, ‘FGF23’, ‘hepcidin’, ‘gut microbiota’, ‘zinc’, and ‘cell’ signal potential therapeutic avenues.

**Figure 7.**
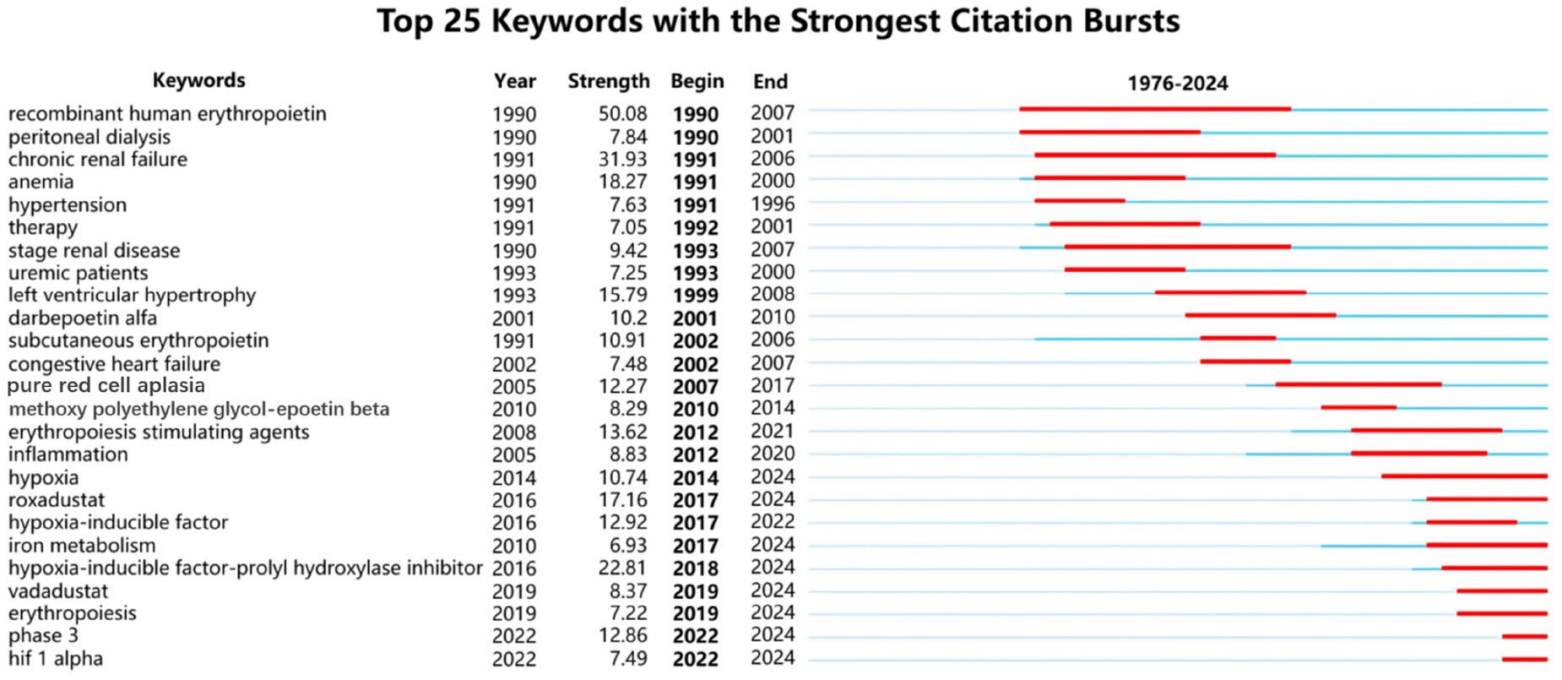
Top 25 keywords exhibiting the strongest citation bursts. The thick blue line represents the keyword’s baseline citation period, while the thick red line represents its citation burst period.

Figure 7 delineates the 25 keywords with the strongest citation bursts. ‘rHuEpo’ demonstrated the longest academic prominence (1990–2007) and the highest burst intensity (50.08), solidifying its foundational role in therapeutic development. Sequential transitions through ‘darbepoetin alfa’ (2001–2010), ‘methoxy polyethylene glycol-epoetin beta’ (2010–2014), and ‘ESAs’ (2012–2021) reflect sustained scholarly attention to EPO-based therapies. Emerging bursts for ‘roxadustat’ (2017–2024), ‘HIF-PHIs’ (2018–2024), ‘vadadustat’ (2019–2024), and ‘phase 3’ (2022–2024) underscore HIF-PHIs as the current research hotspot, with pivotal phase III trial outcomes poised to reshape clinical guidelines. Concurrently, persistent bursts for ‘inflammation’ (2012–2020), ‘hypoxia’ (2014–2024), ‘iron metabolism’ (2017–2024), ‘erythropoiesis’ (2019–2024), and ‘HIF-1α’ (2022–2024) confirm an enduring focus on mechanistic investigations.

## 4. Discussion

In this work, we employed bibliometric methodologies to characterize the dynamic, diverse, and evolving academic landscape of renal anemia using WOSCC data.

Publication trends reveal three evolutionary phases (1976–2024). The nascent phase (1976–1989) coincided with foundational EPO biology discoveries, culminating in the identification of renal oxygen-sensing mechanisms [45]. The subsequent fluctuating growth phase (1990–2017) aligned with clinical adoption of rHuEpo and its derivatives, alongside guideline refinements—reflecting cyclical patterns of therapeutic optimization and risk-benefit debates [24,46]. The rapid surge post-2018 correlates with rising CKD epidemiological burden and therapeutic breakthroughs in HIF-PHIs, exemplified by roxadustat’s approval [4,47]. This acceleration underscores renal anemia’s growing priority in global nephrology research.

Globally, renal anemia research exhibits a dynamic balance of multicentric collaboration and regional specialization, structured around three hubs: East Asia (Japan and China), North America (the US), and Europe (Germany, the UK, and Italy). Japan and China have shown remarkable publication growth over the past decade, driven primarily by their leadership in multicenter HIF-PHI clinical trials. Although these nations contributed 39.12% of global publications, their citation impact lags behind that of the US and Germany, highlighting the sustained scientific leadership of Western hubs. The US and Germany act as central collaboration bridges, with the highest centrality and TLS, which correlate strongly with superior average citations per publication. Furthermore, pharmaceutical-academic symbiosis is prevalent, with 80% of the top 10 institutions collaborating with industry leaders. In contrast, European centers like Humboldt University maintain therapeutic innovation largely through public funding, illustrating diversified translational pathways. To enhance their global influence, China and Japan should strategically deepen collaboration with high-centrality hubs via joint trials and shared protocols.

Author analysis identified key academic leaders in the renal anemia research, whose work spans basic science to clinical application. These scholars have played pivotal roles in clinical trials and guideline development for rHuEpo and derivatives, iron therapies, and HIF-PHIs. Among the top 20 authors: Iain C. Macdougall (King’s College Hospital, UK) is the most productive; Francesco Locatelli (Alessandro Manzoni Hospital, Italy) has the highest H-index; Masaomi Nangaku (University of Tokyo, Japan) demonstrates the greatest citation impact and centrality; and Tadao Akizawa (Showa University, Japan) exhibits the highest TLS value. Their contributions have advanced scientific understanding of renal anemia treatment and profoundly influenced global clinical management of CKD patients.

Among the top 20 journals, leading publications such as *Nephrology Dialysis Transplantation*, *Kidney International*, and *American Journal of Kidney Diseases* lead in both publication volume and citation frequency, reflecting their substantial academic scholarly influence. Thus, researchers interested in this field are encouraged to stay updated through these journals and consider submitting their work. Furthermore, our analysis reveals that while renal anemia research remains rooted in nephrology, it increasingly integrates knowledge from hematology, biochemistry, and fundamental medical sciences.

This study systematically delineates the foundational research framework and knowledge network through co-citation analysis. Among the top 20 most co-cited references, clinical trials dominate (85%), followed by reviews (10%) and clinical practice guidelines (5%). This distribution underscores the pivotal role of clinical trials in advancing evidence-based practice, while reviews and guidelines complementarily synthesize theoretical knowledge and inform clinical decision-making.

Based on co-cited references, keyword co-occurrence, and temporal evolution, our bibliometric analysis identified key research domains in renal anemia: pathophysiological mechanisms and clinical therapy advances, particularly the development of ESAs, iron therapy, and HIF-PHIs.

The pathogenesis and progression of renal anemia involve multifactorial mechanisms, a landscape reflected in evolving bibliometric trends. A central feature is the progressive depletion of functional renal EPO-producing cells and the subsequent decline in endogenous EPO synthesis. In CKD, these cells undergo transdifferentiation, mitochondrial dysfunction, or epigenetic silencing of the HIF-2α gene, further suppressing EPO expression [48–52]. This is corroborated by ongoing citation bursts of keywords such as ‘erythropoiesis’ (2019–2024) and ‘HIF-1 alpha’ (2022–2024). Dysregulated iron metabolism, particularly functional iron deficiency driven by hepcidin upregulation, constitutes another key mechanism. Chronic inflammation and oxidative stress in CKD promote cytokine-mediated hepcidin overexpression, which inhibits iron absorption and recycling [5,53–55]. The importance of this pathway is evidenced by persistent citation bursts of ‘iron metabolism’ (2017–2024) and ‘hypoxia’ (2014–2024), as well as the earlier foundational burst of ‘inflammation’ (2012–2020). In recent years, the role of uremic toxins has gained traction, emerging as a new focus in keyword co-occurrence network and cluster analyses. Protein-bound uremic toxins (e.g., indoxyl sulfate) contribute to anemia by impairing erythroid progenitor proliferation, disrupting EPO receptor signaling, increasing oxidative stress, and stimulating hepcidin production [56–60]. Research in this area has shown gradually intensifying momentum since 2018, reflecting deeper insights into toxin accumulation in anemia pathology. Temporal co-citation reference analysis visually maps this scientific consensus, showing how recent research has coalesced around interconnected clusters. While the largest and most recent cluster #0 (HIF-PHI) underscores a therapeutic shift toward HIF modulation, its strong co-citation linkages with emerging clusters #8 (iron homeostasis) and #11 (micro-inflammatory response) indicate that investigations into iron dysregulation and chronic inflammation remain highly active and are intrinsically linked to understanding and treatment of renal anemia. Notably, in-depth uremic toxin research is interweaving with these traditional mechanisms, collectively advancing to a more comprehensive pathological framework. In summary, renal anemia arises from intertwined pathways including impaired EPO production, iron dysregulation, chronic inflammation, oxidative stress, and uremic toxicity. This complexity is now being addressed through multi-targeted therapeutic strategies informed by these converging research fronts.

Keyword citation burst analysis (1990–2021) underscores that ESAs remain the cornerstone of renal anemia management, evidenced by high-frequency keywords such as ‘rHuEPO’, ‘darbepoetin alfa’, ‘ESA’, and ‘epoetin alfa’ in Table S8. This clinical dominance is further supported by highly cited literature; notably, the top five articles in Table S7 are all ESA clinical trials. The sequential sustained prominence of keywords, progressing from ‘rHuEpo’ (1990–2007) to ‘darbepoetin alfa’ (2001–2010), ‘methoxy polyethylene glycol-epoetin beta’ (2010–2014), and ‘ESAs’ (2012–2021), not only reflects continuous ESA innovation but also reveals a shift from short-acting agents (epoetin alfa/beta) to long-acting formulations (darbepoetin alfa, methoxy polyethylene glycol-epoetin beta) [61,62]. The emergence of biosimilars such as HX575 and epoetin zeta in keyword cluster analysis indicates the growing focus on cost-effective alternatives [63,64]. However, bibliometric data also reveal ESA limitations. The co-occurrence of ‘ESAs’, ‘anti-erythropoietin antibody’ and ‘pure red cell aplasia (PRCA)’ in the keyword cluster #7 analysis indicates antibody-mediated ESA risk, although this rare but serious complication is mainly caused by subcutaneous ESA administration [65]. Keyword ‘PRCA’ demonstrates significant citation bursts during 2007–2017 (strength: 12.27), directly validating the peak period of antibody-mediated PRCA risk. Keywords like ‘mortality’, ‘inflammation’, and ‘iron deficiency’ are also associated with cardiovascular risks and treatment hyporesponsiveness [66]. Future studies should address ESA hyporesponsiveness and evaluate the long-term cardiovascular safety of newer ESAs and biosimilars.

Iron supplementation emerges as a persistent and central research theme in renal anemia management, underscored by multi-method bibliometric evidence. CiteSpace identifies a dedicated cluster #7 (iron supplementation), which emerged prominently from the early 1990s to the early 2000s and remained a key focus throughout this period. Keyword analysis ranks ‘iron’, ‘intravenous iron supplementation’, and ‘iron deficiency’ among the top 30 high-frequency keywords. In the VOSviewer co-occurrence network, ‘iron supplementation’ exhibits strong connections to ‘renal anemia’, ‘EPO’, and ‘CKD’. The co-occurrence of ‘oral iron’, ‘intravenous iron therapy’, and multiple iron formulations (ranging from traditional agents like iron dextran to newer ones such as ferric carboxymaltose and ferumoxytol) in keyword cluster #4 analysis not only uncovers the dual-pathway characteristic of iron supplementation research in renal anemia but also reflects the innovation of iron treatment strategies. Recent shifts in frontiers are revealed by citation burst analysis: ‘iron metabolism’ (2017–2024) highlights growing mechanistic interest in iron homeostasis; ‘HIF-PHI’ (2018–2024) signals exploration of HIF signaling in iron regulation [67–69]. Together, these bibliometric insights collectively demonstrate iron supplementation’s enduring centrality in renal anemia research, spanning foundational efficacy, mechanistic exploration, and clinical optimization. Critically, the recent UK Kidney Association Clinical Practice Guideline emphasizes iron repletion prior to ESA/HIF-PHI therapy and that treatment selection must balance efficacy, tolerability, and risks [70].

The bibliometric landscape since 2017 definitively establishes HIF-PHIs as a dominant emerging field in renal anemia research, marking a paradigm shift from exogenous EPO replacement to physiological hypoxia-response modulation. This trajectory is confirmed by CiteSpace cluster analysis, which identifies cluster #0 (HIF-PHI) as the largest and most recent research hub. High-frequency keywords (roxadustat, HIF-PHI, and HIF) in Table S8 further validate their significance. Mechanistic synergy, characterized by dual activation of endogenous EPO production and enhanced iron bioavailability [71], is empirically supported by robust co-citation bonds between cluster #0 (HIF-PHI) and cluster #8 (iron homeostasis), illustrating interdisciplinary integration of hypoxia-response pathways. HIF-PHIs’ particular benefit in ESA-hyporesponsive patients and those with inflammatory comorbidities [72–75] is supported by co-citation bonds between clusters #0 and #11 (micro-inflammatory response). Keywords ‘roxadustat’ (2017–2024), ‘HIF-PHIs’ (2018–2024), ‘vadadustat’ (2019–2024), and ‘phase 3’ (2022–2024) exhibit the strongest bursts, emphasizing rigorous evaluation of late-stage clinical outcomes. Clinical translation is evidenced by global approvals of six oral agents (roxadustat, daprodustat, vadadustat, molidustat, enarodustat, and desidustat) since 2018. Despite therapeutic promise, safety concerns including thromboembolism and cardiovascular events drive regulatory divergence, exemplified by the FDA’s 2021 rejection of roxadustat and restricted approval of daprodustat for dialysis-dependent patients [11]. Further, the pleiotropic nature of HIF-PHIs introduces additional clinical challenges: their broad regulatory effects on angiogenesis, glucose metabolism, and cellular proliferation may exacerbate retinal pathologies, malignant progression, polycystic kidney disease manifestations, and pulmonary hypertension [8,10]. A single-center retrospective cohort study also reported that roxadustat was associated with reversible central hypothyroidism in hemodialysis patients [76]. The latest UK Kidney Association Clinical Practice Guideline advises cautious use in high-risk groups and emphasizes post-marketing surveillance [70]. Bibliometric analysis concurrently exposes three critical evidence gaps: (1) scarcity of ESA-to-HIF-PHI dose conversion data; (2) limited safety profiles for ESA/HIF-PHI co-administration; and (3) inadequate head-to-head trials among HIF-PHIs. Future priorities require addressing these gaps through biomarker-guided patient stratification, particularly for high-risk subpopulations.

Beyond established therapies such as ESAs, iron supplementation, and HIF-PHIs, temporal keyword analysis and emerging thematic clusters point to several potential avenues, warranting further investigation in renal anemia research: (1) SGLT2 inhibitors (e.g., dapagliflozin, canagliflozin, tofogliflozin) ameliorate anemia in diabetic and non-diabetic CKD via hepcidin suppression, improved iron availability, EPO stimulation, gut microbiome modulation, and anti-inflammatory effects [77–84]. However, luseogliflozin did not show similar benefits in a non-diabetic animal model [85]. (2) Hepcidin antagonists aim to counter pathological hepcidin overexpression in CKD. Promising agents include the anti-BMP6 antibody LY3113593 [86], the anticalin PRS-080 [87], and modulators of endogenous signaling such as fibrinogen-like protein 1 [88]. (3) FGF23 signaling modulators: Elevated FGF23 is linked to anemia and iron deficiency in CKD [89,90]. Preclinical studies show FGF23 inhibition improves erythropoiesis and iron metabolism [91], and phosphate control may reduce FGF23 and improve anemia [92,93], though balancing mineral homeostasis remains challenging [94]. (4) Gut microbiota modulation: Dysbiosis contributes to inflammation, toxin accumulation, and ESA hyporesponsiveness [95]. Probiotics, prebiotics, and dietary interventions may improve hemoglobin in ESRD and hemodialysis patients [96–98], though large-scale trials are needed. 5) Zinc supplementation: Zinc demonstrates erythropoietic effects, reduces inflammation, and may improve survival in hemodialysis patients [99–101]. Co-administration with HIF-PHIs might alleviate copper overload, and zinc acetate hydrate can reduce ESA requirements, but dosing must be cautious to avoid copper deficiency [102,103]. 6) Cell therapy: iPSC-derived EPO-producing cells show promise in CKD models [104,105], but issues of immune compatibility and cell viability require further study.

## 5. Limitations

This study has several limitations that should be acknowledged. Firstly, the literature search was restricted to English-language publications, which may underrepresent region-specific therapeutic approaches (e.g., traditional Chinese medicine interventions documented in Chinese academic databases). Secondly, our analysis was confined to the WOSCC primarily for bibliometric software compatibility reasons. This may result in selection bias, as significant publications indexed exclusively in other databases (e.g., Scopus) were not included. Future studies could mitigate this by employing a multi-database search strategy. Thirdly, our data retrieval was conducted on 6 November 2024. While this captures the foundational and established body of knowledge in the field of renal anemia up to that point, it may not reflect the very latest research trends or emerging publications in early- to mid-2025. However, the core intellectual structure, major themes, and historical evolution identified herein are expected to remain robust and informative. Future studies could build upon this baseline by incorporating more recent publications to track the dynamic development of the field.

## 6. Conclusions

As one of the first comprehensive analyses of renal anemia, this study constructs a knowledge framework that maps research trends, collaboration networks, current hotspots, and emerging frontiers in the field. The findings indicate that global research has primarily focused on elucidating disease mechanisms and refining therapeutic strategies. Current research efforts are concentrated on ESAs, iron supplementation, and HIF-PHIs. Emerging approaches, including SGLT2 inhibitors, hepcidin antagonists, FGF23 signaling modulators, gut microbiota regulators, zinc supplementation, and stem cell-derived therapies, exhibit therapeutic potential in early-stage studies. Future investigations should prioritize three key directions: (1) developing personalized treatment protocols, particularly combination therapies targeting multifactorial pathophysiology; (2) discovering new drug candidates through mechanistic studies; and (3) establishing robust pharmacovigilance systems for newly approved agents. These conclusions provide valuable insights for guiding clinical practice and shaping future renal anemia research.

## Supplementary Material

Supplementary Materials.docx

## Data Availability

Data will be made available on request.
