# Supplementary material for: Global research trends in renal anemia: a multidimensional bibliometric study
Source: Ren Fail. 2025 Nov 4;47(1):2580457. doi: 10.1080/0886022X.2025.2580457 (PMC12587798; doi:10.1080/0886022X.2025.2580457)
Supplement: Supplementary Materials.docx [file IRNF_A_2580457_SM1160.docx]

Supplementary Materials

**Global research trends in renal anemia: a multidimensional bibliometric study**

Yuanchen Niu^a,b*^, Yufang Wang^c*^, Changhong Huo^c^ and Yi Fang^a,b,d^

^a^Key Laboratory of Molecular Target & Clinical Pharmacology and the State Key Laboratory of Respiratory Disease, School of Pharmaceutical Sciences & the Fifth Affiliated Hospital, Guangzhou Medical University, Guangzhou, China; ^b^Phase I Clinical Research Center, Key Laboratory of Biological Targeting Diagnosis, Therapy and Rehabilitation of Guangdong Higher Education Institutes, The Fifth Affiliated Hospital of Guangzhou Medical University, Guangzhou, China; ^c^School of Pharmaceutical Sciences, Hebei Medical University, Shijiazhuang, China; ^d^Clinical Trial Institution Research Ward, Peking University People’s Hospital, Beijing, China

**List of contents**

Figure S1: Study flowchart showing the literature retrieval strategy and the process of literature exclusion.

Figure S2: Research methodology flowchart for the bibliometric analysis.

Table S1. Detailed search strategy executed in the Web of Science Core Collection​.

Table S2. The tasks executed by the software.

Table S3: Top 10 productive countries and institutions.

Table S4: Top 10 productive authors.

Table S5: Top 20 journals with the most publications related to renal anemia.

Table S6: Top 20 co-cited journals related to renal anemia research.

Table S7: Top 20 co-cited articles related to renal anemia research

Table S8: Top 30 keywords in renal anemia research literature.


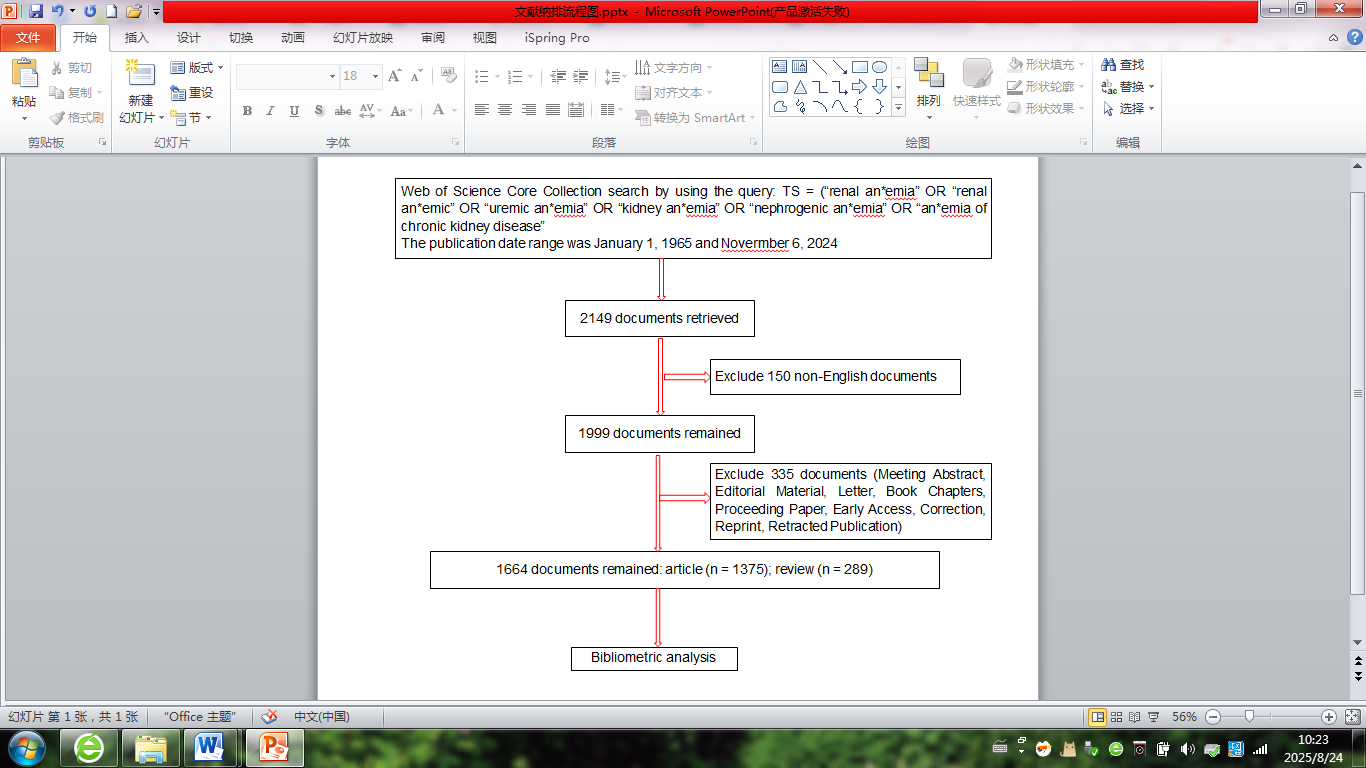


**Figure S1: Study flowchart showing the literature retrieval strategy and the process of literature exclusion.**


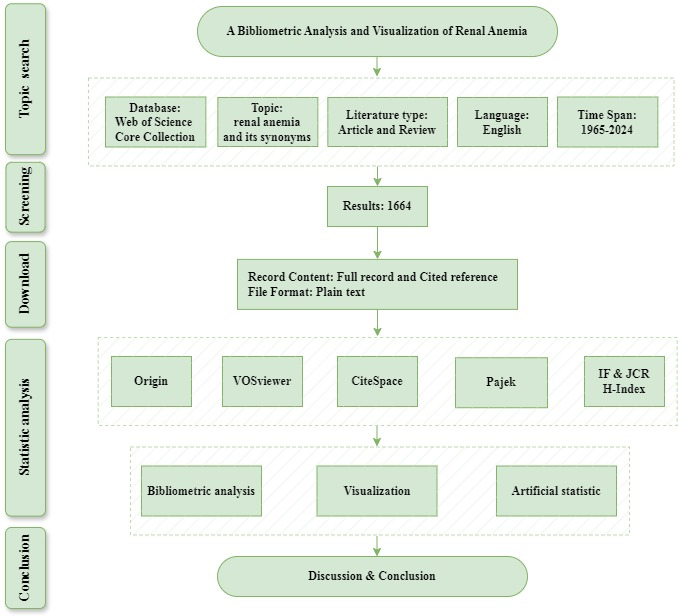


**Figure S2: Research methodology flowchart for the bibliometric analysis.**

**Table S1. Detailed search strategy executed in the Web of Science Core Collection.**

| Concept | Search Terms | Boolean operator |
| --- | --- | --- |
| renal anemia | “renal anemia”  “renal anaemia” | OR |
|  | “renal anemic”  “renal anaemic” |  |
|  | “uremic anemia”  “uremic anaemia” |  |
|  | “kidney anemia”  “kidney anaemia” |  |
|  | “nephrogenic anemia”  “nephrogenic anaemia”  “anemia of chronic kidney disease”  “anaemia of chronic kidney disease” |  |

Note: Final Combined Query: TS = (“renal an*emia” OR “renal an*emic” OR “uremic an*emia” OR “kidney an*emia” OR “nephrogenic an*emia” OR “an*emia of chronic kidney disease”. The asterisk (*) serves as a wildcard character to capture both spelling variations: "anemia" and "anaemia".

**Table S2. The tasks executed by the software.**

| Tool | task | Figure |
| --- | --- | --- |
| VOSviewer | collaboration networks | 2B, 2C, 2D |
| VOSviewer + Pajek | cluster analysis and visualizing the temporal evolution of keyword co-occurrence networks | 6A, 6B |
| CiteSpace | co-cited reference analysis, citation burst detection of references and keywords, and dual-map overlays | 3, 4A, 4B, 5, 7 |
| Origin | plot publication trends | 1, 2A |

**Table S3: Top 10 productive countries and institutions.**

| Rank | Country | Publications | Citations | Average citations | H-index | Centrality | TLS | Rank | Institution | Publications | Citations | Average citations | Centrality | TLS |
| --- | --- | --- | --- | --- | --- | --- | --- | --- | --- | --- | --- | --- | --- | --- |
| 1 | Japan | 407 | 8,158 | 20.04 | 41 | 0.09 | 66 | 1 | Showa University (Japan) | 33 | 684 | 20.73 | 0.02 | 82 |
| 2 | USA | 278 | 10,433 | 37.53 | 152 | 0.26 | 260 | 2 | University of Tokyo (Japan) | 31 | 2,003 | 64.61 | 0.07 | 57 |
| 3 | China | 244 | 2,516 | 10.31 | 42 | 0.01 | 40 | 3 | King's College Hospital (UK) | 28 | 743 | 26.54 | 0.03 | 24 |
| 4 | Germany | 197 | 7,001 | 35.54 | 74 | 0.17 | 234 | 4 | Tohoku University (Japan) | 26 | 897 | 34.5 | 0.04 | 36 |
| 5 | UK | 149 | 4,513 | 30.29 | 78 | 0.19 | 229 | 5 | Jikei University (Japan) | 25 | 808 | 32.32 | 0.01 | 41 |
| 6 | Italy | 103 | 2,653 | 25.76 | 83 | 0.10 | 168 | 6 | Tokyo Women's Medical University (Japan) | 24 | 398 | 16.58 | 0.01 | 37 |
| 7 | Austria | 62 | 1,774 | 28.61 | 75 | 0.01 | 75 | 7 | Kyoto University (Japan) | 22 | 922 | 41.91 | 0.01 | 33 |
| 8 | Spain | 55 | 1,147 | 20.85 | 61 | 0.04 | 91 | 8 | Humboldt university (Germany) | 21 | 661 | 31.48 | 0.02 | 13 |
| 9 | Australia | 51 | 1,337 | 26.22 | 69 | 0.08 | 71 | 9 | University Hospital Vienna (Austria) | 18 | 501 | 27.83 | 0.01 | 17 |
| 10 | Sweden | 51 | 1,406 | 27.57 | 73 | 0.07 | 71 | 10 | University of Vienna (Austria) | 17 | 613 | 36.06 | 0.01 | 1 |

**Table S4: Top 10 productive authors.**

| Rank | Author | Publications | Citations | Average citations | Centrality | TLS | Country | Instituent | H-Index | |
| --- | --- | --- | --- | --- | --- | --- | --- | --- | --- | --- |
| 1 | Iain C Macdougall | 38 | 1,701 | 44.76 | 0.02 | 51 | UK | King's College Hospital | | 65 |
| 2 | Tadao Akizawa | 29 | 633 | 21.83 | 0.02 | 75 | Japan | Showa University | | 58 |
| 3 | Masaomi Nangaku | 28 | 1,826 | 65.21 | 0.03 | 57 | Japan | University of Tokyo | | 58 |
| 4 | Francesco Locatelli | 28 | 787 | 28.11 | 0.01 | 28 | Italy | Alessandro Manzoni Hospital | | 90 |
| 5 | Tetsuhiro Tanaka | 18 | 627 | 34.83 | 0 | 30 | Japan | University of Tokyo | | 38 |
| 6 | Hannelore Hampl | 17 | 365 | 21.47 | 0 | 38 | Germany | Humboldt University of Berlin | | 20 |
| 7 | Walter H Hörl | 15 | 457 | 30.47 | 0 | 9 | Austria | University Hospital Vienna | | 60 |
| 8 | Takahiro Kuragano | 15 | 274 | 18.27 | 0 | 32 | Japan | Hyogo Medical University | | 20 |
| 9 | Takeshi Nakanishi | 14 | 274 | 19.57 | 0 | 32 | Japan | Osaka Metropolitan University | | 26 |
| 10 | Hiroyasu Yamamoto | 14 | 379 | 27.07 | 0.02 | 55 | Japan | Jikei University | | 16 |

**Table S5: Top 20 journals with the most publications related to renal anemia.**

| **Rank** | **Publications** | **% of Total Publication** | **Journal** | | | |
| --- | --- | --- | --- | --- | --- | --- |
|  |  |  | **Name** | **JCR** | **IF** | **H-Index** |
| 1 | 116 | 6.97 | Nephrology Dialysis Transplantation | Q1 | 4.8 | 197 |
| 2 | 61 | 3.67 | Clinical Nephrology | Q3 | 1.1 | 82 |
| 3 | 42 | 2.52 | Kidney International | Q1 | 14.8 | 318 |
| 4 | 41 | 2.46 | American Journal of Kidney Diseases | Q1 | 9.4 | 240 |
| 5 | 38 | 2.28 | Nephron | Q2 | 2.3 | 99 |
| 6 | 35 | 2.1 | Therapeutic Apheresis and Dialysis | Q3 | 1.5 | 60 |
| 7 | 31 | 1.86 | Clinical and Experimental Nephrology | Q2 | 2.2 | 63 |
| 8 | 28 | 1.68 | BMC Nephrology | Q2 | 2.2 | 75 |
| 9 | 27 | 1.62 | Blood Purification | Q2 | 2.2 | 67 |
| 10 | 26 | 1.56 | American Journal of Nephrology | Q1 | 4.3 | 103 |
| 11 | 26 | 1.56 | Nephrology | Q2 | 2.4 | 75 |
| 12 | 24 | 1.44 | Renal Failure | Q1 | 3 | 65 |
| 13 | 22 | 1.32 | International Urology and Nephrology | Q3 | 1.8 | 63 |
| 14 | 20 | 1.2 | PLoS ONE | Q1 | 2.9 | 435 |
| 15 | 19 | 1.14 | Journal of Nephrology | Q2 | 2.7 | 79 |
| 16 | 19 | 1.14 | Journal of the American Society of Nephrology | Q1 | 10.3 | 321 |
| 17 | 15 | 0.9 | Clinical Kidney Journal | Q1 | 3.9 | 64 |
| 18 | 15 | 0.9 | Current Opinion in Nephrology and Hypertension | Q2 | 2.2 | 105 |
| 19 | 15 | 0.9 | Pediatric Nephrology | Q1 | 2.6 | 124 |
| 20 | 14 | 0.84 | International Journal of Artificial Organs | Q3 | 1.4 | 64 |

**Table S6: Top 20 co-cited journals related to renal anemia research.**

| **Rank** | **Citations** | **Journal** | | | |
| --- | --- | --- | --- | --- | --- |
|  |  | **Name** | **JCR** | **IF** | **H-Index** |
| 1 | 4,230 | Nephrology Dialysis Transplantation | Q1 | 4.8 | 197 |
| 2 | 3,984 | Kidney International | Q1 | 14.8 | 318 |
| 3 | 3,506 | American Journal of Kidney Diseases | Q1 | 9.4 | 240 |
| 4 | 3,367 | Journal of the American Society of Nephrology | Q1 | 10.3 | 321 |
| 5 | 2,705 | New England Journal of Medicine | Q1 | 96.2 | 1184 |
| 6 | 1,783 | Blood | Q1 | 21 | 525 |
| 7 | 1,104 | Clinical journal of the American Society of Nephrology | Q1 | 8.5 | 189 |
| 8 | 1,092 | Nephron | Q2 | 2.3 | 99 |
| 9 | 1,001 | Clinical Nephrology | Q3 | 1.1 | 82 |
| 10 | 957 | Lancet | Q1 | 98.4 | 895 |
| 11 | 947 | American Journal of Nephrology | Q1 | 4.3 | 103 |
| 12 | 900 | Journal of Clinical Investigation | Q1 | 13.3 | 544 |
| 13 | 826 | Journal of Biological Chemistry | Q2 | 4 | 556 |
| 14 | 777 | Proceedings of the National Academy of Sciences of the United States of America | Q1 | 9.4 | 869 |
| 15 | 616 | PLoS ONE | Q1 | 2.9 | 435 |
| 16 | 694 | Circulation | Q1 | 35.5 | 674 |
| 17 | 484 | Contributions to Nephrology | - | - | 60 |
| 18 | 440 | Therapeutic Apheresis and Dialysis | Q3 | 1.5 | 60 |
| 19 | 423 | Annals of Internal Medicine | Q1 | 19.6 | 433 |
| 20 | 386 | Science | Q1 | 44.7 | 1336 |

**Table S7: Top 20 co-cited articles related to renal anemia research.**

| **Rank** | **Title** | **Year** | **Journal** | **Type of research** | **Citations** |
| --- | --- | --- | --- | --- | --- |
| 1 | Correction of anemia with epoetin alfa in chronic kidney disease (DOI: 10.1056/NEJMoa065485) | 2006 | New England Journal of Medicine | Clinical Trial | 241 |
| 2 | A trial of darbepoetin alfa in type 2 diabetes and chronic kidney disease (DOI: 10.1056/NEJMoa0907845) | 2009 | New England Journal of Medicine | Clinical Trial | 198 |
| 3 | Correction of the anemia of end-stage renal disease with recombinant human erythropoietin. Results of a combined phase I and II clinical trial (DOI: 10.1056/NEJM198701083160203) | 1987 | New England Journal of Medicine | Clinical Trial | 180 |
| 4 | Normalization of hemoglobin level in patients with chronic kidney disease and anemia (DOI: 10.1056/NEJMoa062276) | 2006 | New England Journal of Medicine | Clinical Trial | 178 |
| 5 | The effects of normal as compared with low hematocrit values in patients with cardiac disease who are receiving hemodialysis and epoetin (DOI: 10.1056/NEJM199808273390903) | 1998 | New England Journal of Medicine | Clinical Trial | 176 |
| 6 | Roxadustat treatment for anemia in patients undergoing long-term dialysis (DOI: 10.1056/NEJMoa1901713) | 2019 | New England Journal of Medicine | Clinical Trial | 133 |
| 7 | Effect of human erythropoietin derived from recombinant DNA on the anaemia of patients maintained by chronic haemodialysis (DOI: 10.1016/S0140-6736(86)92192-6) | 1986 | Lancet | Clinical Trial | 130 |
| 8 | Mechanisms of anemia in CKD (DOI: 10.1681/ASN.2011111078) | 2012 | Journal of the American Society of Nephrology | Review | 128 |
| 9 | Roxadustat for anemia in patients with kidney disease not receiving dialysis (DOI: 10.1056/NEJMoa1813599) | 2019 | New England Journal of Medicine | Clinical Trial | 112 |
| 10 | Recombinant human erythropoietin in anemic patients with end-stage renal disease. Results of a phase III multicenter clinical trial (DOI: 10.7326/0003-4819-111-12-992) | 1989 | Annals of Internal Medicine | Clinical Trial | 91 |
| 11 | Secondary analysis of the CHOIR trial epoetin-α dose and achieved hemoglobin outcomes (DOI: 10.1038/ki.2008.295) | 2008 | Kidney International | Clinical Trial | 83 |
| 12 | Roxadustat (FG-4592) versus epoetin alfa for anemia in patients receiving maintenance hemodialysis: A phase 2, randomized, 6-to 19-week, open-label, active-comparator, dose-ranging, safety and exploratory efficacy study (DOI: 10.1053/j.ajkd.2015.12.020) | 2016 | American Journal of Kidney Diseases | Clinical Trial | 82 |
| 13 | Hypoxia-inducible factor prolyl hydroxylase inhibitors: A potential new treatment for anemia in patients with CKD (DOI: 10.1053/j.ajkd.2016.12.011) | 2017 | American Journal of Kidney Diseases | Review | 81 |
| 14 | KDOQI clinical practice guidelines and clinical practice recommendations for anemia in chronic kidney disease (DOI: 10.1053/j.ajkd.2006.03.010) | 2006 | American Journal of Kidney Diseases | Practice Guideline | 81 |
| 15 | Erythropoietic response and outcomes in kidney disease and type 2 diabetes (DOI: 10.1056/nejmoa1005109) | 2010 | New England Journal of Medicine | Clinical Trial | 79 |
| 16 | Randomized placebo-controlled dose-ranging and pharmacodynamics study of roxadustat (FG-4592) to treat anemia in nondialysis-dependent chronic kidney disease (NDD-CKD) patients (DOI: 10.1093/ndt/gfv302) | 2015 | Nephrology Dialysis Transplantation | Clinical Trial | 76 |
| 17 | Roxadustat (FG-4592): Correction of anemia in incident dialysis patients (DOI: 10.1681/asn.2015030241) | 2016 | Journal of the American Society of Nephrology | Clinical Trial | 71 |
| 18 | Phase 3, randomized, double-blind, active-comparator (darbepoetin alfa) study of oral roxadustat in CKD patients with anemia on hemodialysis in Japan (DOI: 10.1681/asn.2019060623) | 2020 | Journal of the American Society of Nephrology | Clinical Trial | 68 |
| 19 | Four-week studies of oral hypoxia-inducible factor-prolyl hydroxylase inhibitor GSK1278863 for treatment of anemia (DOI: 10.1681/asn.2014111139) | 2016 | Journal of the American Society of Nephrology | Clinical Trial | 67 |
| 20 | The impact of anemia on cardiomyopathy, morbidity, and mortality in end-stage renal disease (DOI: 10.1016/s0272-6386(96)90130-4) | 1996 | American Journal of Kidney Diseases | Comparative Study | 67 |

**Table S8: Top 30 keywords in renal anemia research literature.**

| **Rank** | **keyword** | **occurrences** | **TLS** | **Rank** | **keyword** | **occurrences** | **TLS** |
| --- | --- | --- | --- | --- | --- | --- | --- |
| 1 | anemia | 736 | 5275 | 16 | ESRD | 140 | 1061 |
| 2 | hemodialysis | 667 | 4902 | 17 | HIF-PHI | 124 | 935 |
| 3 | CKD | 615 | 4801 | 18 | inflammation | 119 | 891 |
| 4 | renal anemia | 549 | 3558 | 19 | HIF | 110 | 750 |
| 5 | EPO | 531 | 3533 | 20 | hepcidin | 105 | 826 |
| 6 | rHuEPO | 392 | 2777 | 21 | clinical trial | 104 | 838 |
| 7 | dialysis | 273 | 2220 | 22 | Intravenous iron supplementation | 93 | 832 |
| 8 | chronic renal failure | 223 | 1643 | 23 | peritoneal dialysis | 91 | 628 |
| 9 | darbepoetin alfa | 194 | 1634 | 24 | quality of life | 85 | 761 |
| 10 | ESA | 191 | 1718 | 25 | oxidative stress | 85 | 665 |
| 11 | mortality | 179 | 1424 | 26 | hypoxia | 79 | 533 |
| 12 | epoetin alfa | 178 | 1530 | 27 | safety | 73 | 601 |
| 13 | roxadustat | 165 | 1222 | 28 | epoetin | 73 | 592 |
| 14 | iron | 162 | 1324 | 29 | iron deficiency | 73 | 574 |
| 15 | hemoglobin | 142 | 1123 | 30 | heart failure | 71 | 576 |
